# Supplementary material for: H3K27me3 chromatin heterogeneity reveals variable cell responses to estrogen and endocrine treatment
Source: bioRxiv. 2025 Sep 20:2025.09.18.677184. Preprint. [Version 1] doi: 10.1101/2025.09.18.677184 (PMC12458927; doi:10.1101/2025.09.18.677184)
Supplement: 1 [file NIHPP2025.09.18.677184V1-supplement-1.pdf]

bioRxiv preprint doi: https://doi.org/10.1101/2025.09.18.677184; this version posted September 20, 2025. The copyright holder for this preprint (which was not certified by peer review) is the author/funder. This article is a US Government work. It is not subject to copyright under 17 USC 105 and is also made available for use under a CC0 license.

Supplementary material

Table S1. Modeling rates

	1/days	95CI
lambda:	0,290899	0.256926 - 0.335398
Aeq:	0,179115	0.171807 - 0.186465
kon:	0,0521043	0.045866 - 0.059653
koff:	0,238795	0.210739 - 0.276273

Supplementary Figure 1.

A. Histogram of TFF1 sorting strategy
B. Volcano plot of Nascent-RNAseq differentially expressed genes between TFF1^{high} and TFF1^{low} cells. The yellow dots represent significantly upregulated genes, the blue dots represent significantly downregulated genes (log2 FC ≥ 1 and adj p < 0.05), and the grey dots represent insignificant differentially expressed.
C. Barplot representing RPKM values of TFF1 expression in Nascent RNAseq in TFF1^{high} compared to TFF1^{low} cells, shows significantly enrichment in TFF1^{high} cells compared to TFF1^{low} cells.

Supplementary Figure 2.

A. Heatmaps of ERa binding in TFF1^{high} and TFF1^{low} cells.
B. Table summarizing the total number of ERα CUT&Tag peaks, differentially bound regions and associated percentages of differential regions in TFF1^{high} and TFF1^{low} cells.
C. Genome browser view of ERα CUT&Tag shows an example of unchanged ERα binding in TFF1^{high} cells and to TFF1^{low} cells.
D. Genome browser view of ERα CUT&Tag shows an example of enriched ERα binding in TFF1^{high} cells compared to TFF1^{low} cells.
E. Genome browser view of ERα CUT&Tag shows an example of enriched ERα binding in TFF1^{low} cells compared to TFF1^{high} cells.

Supplementary Figure 3.

A. Tiles of high TFF1 expressing TFF1-MS2 cells using live-cell imaging after sorting over 14.2 hours.
B. Tiles of low TFF1 expressing TFF1-MS2 cells using live-cell imaging after sorting over 14.2 hours.

bioRxiv preprint doi: https://doi.org/10.1101/2025.09.18.677184; this version posted September 20, 2025. The copyright holder for this preprint (which was not certified by peer review) is the author/funder. This article is a US Government work. It is not subject to copyright under 17 USC 105 and is also made available for use under a CC0 license.

Supplementary Figure 4.

A. Quantification of average number of TFF1 mRNA molecules/cell plotted at days 4, 6 and 10 post sorting in TFF1^{high} and TFF1^{low} cells.
B. Percentage of TFF1 transcribing cells in unsorted cell population stimulated with 1nm E2.
C. Equilibrium model reveals that the percentage of TFF1 transcribing cells from TFF1^{high} and TFF1^{low} cell populations reach an equilibrium at 17.9% after 20 days.
D. Table of statistical analysis showing TFF1 transcription dynamics in TFF1^{high} and TFF1^{low} cells. Comparisons have been made between TFF1^{high} and TFF1^{low} cells over the 10 days-time course after sorting (top table), and between individual days post-sorting (D4 vs D6; D4 vs D10; D6 vs D10) in TFF1^{high} (middle table) and TFF1^{low} cells (bottom table).

Supplementary Figure 5.

A. Genome browser view of TFF1 locus in another set of Nascent RNAseq (triplicates) confirms that transcription at TFF1 locus in increased in TFF1^{high} compared to TFF1^{low} cells.
B. Venn Diagram of overlapping DEGs from the 2 sets of Nascent RNAseq experiments (each set is composed of biological triplicates) shows 1089 common DEGs. Those 1089 DEGs have been used for Gene Ontology analysis.
C. Talbe of the subset of 47 estrogen responsive genes upregulated in TFF1^{high} cells.
D. Volcano plot of RNAseq DEGs between TFF1^{high} and TFF1^{low} cells. The yellow dots represent significantly upregulated genes, the blue dots represent significantly downregulated genes (log2 FC $\geq$ 1 and adj $p < 0.05$), and the grey dots represent insignificant differentially expressed. TFF1 is the most upregulated gene.
E. Venn Diagram representing the distribution of estrogen responsive genes enriched in TFF1^{high} cells (yellow), enriched in TFF1^{low} cells (blue), or unchanged between the two cell populations (grey).

Supplementary Figure 6.

EnrichR gene ontology results for the 934 unique genes with differential H3K27me3 deposition between TFF1^{high} and TFF1^{low} cells using different databases:

A. ENCODE Histone Modifications 2015 database shows an enrichment of H3K27me3 deposition on those genes in different cell lines
B. ChEA Transcription Factor Targets 2022 database shows an enrichment of Polycomb (PRC2) group members binding to those genes
C. Hallmark Msig Database 2020 shows an enrichment of Estrogen Response categories.

bioRxiv preprint doi: https://doi.org/10.1101/2025.09.18.677184; this version posted September 20, 2025. The copyright holder for this preprint (which was not certified by peer review) is the author/funder. This article is a US Government work. It is not subject to copyright under 17 USC 105 and is also made available for use under a CC0 license.

D. Genome browser view of an example of an E2 responsive gene (BAMBI) showing differential H3K27me3 deposition and differential gene expression in $TFF1^{high}$ and $TFF1^{low}$ cells.

E. Genome browser view of an example of a not-E2 responsive gene (c14orf132) showing differential H3K27me3 deposition and differential gene expression in $TFF1^{high}$ and $TFF1^{low}$ cells.

bioRxiv preprint doi: https://doi.org/10.1101/2025.09.18.677184; this version posted September 20, 2025. The copyright holder for this preprint (which was not certified by peer review) is the author/funder. This article is a US Government work. It is not subject to copyright under 17 USC 105 and is also made available for use under a CC0 license.

Supplementary Figure 1

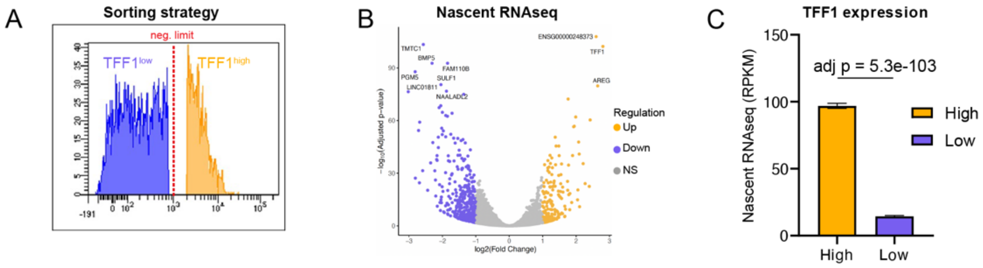

bioRxiv preprint doi: https://doi.org/10.1101/2025.09.18.677184; this version posted September 20, 2025. The copyright holder for this preprint (which was not certified by peer review) is the author/funder. This article is a US Government work. It is not subject to copyright under 17 USC 105 and is also made available for use under a CC0 license.

Supplementary Figure 2

A

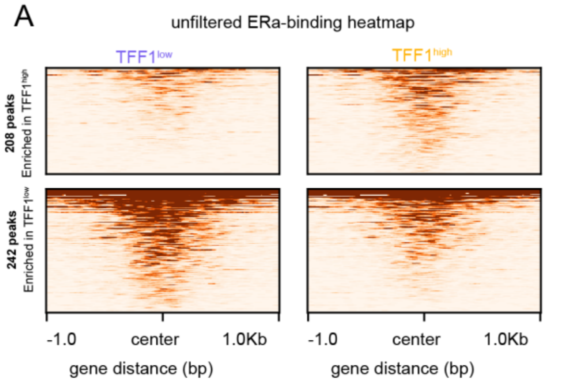

B

Sorted population	Total ERα peaks called	Number of differential regions (FDR < 0.05)	Percentage
TFF1 high	10982	208	1.9
TFF1 low	11947	242	2

C

D

E

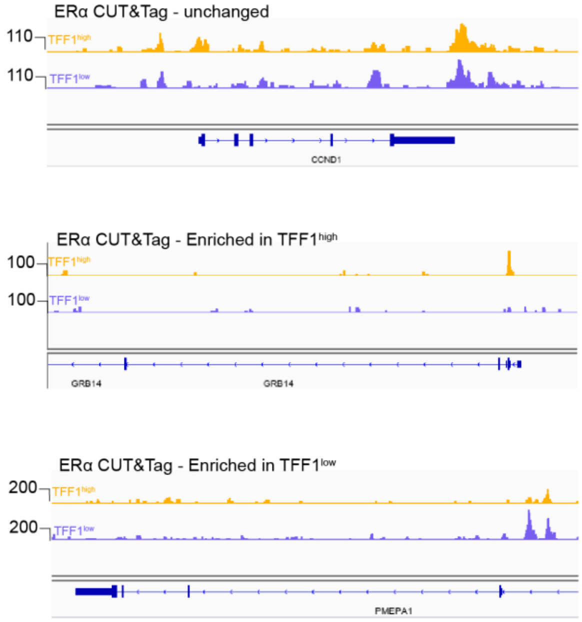

bioRxiv preprint doi: https://doi.org/10.1101/2025.09.18.677184; this version posted September 20, 2025. The copyright holder for this preprint (which was not certified by peer review) is the author/funder. This article is a US Government work. It is not subject to copyright under 17 USC 105 and is also made available for use under a CC0 license.

Supplementary Figure 3

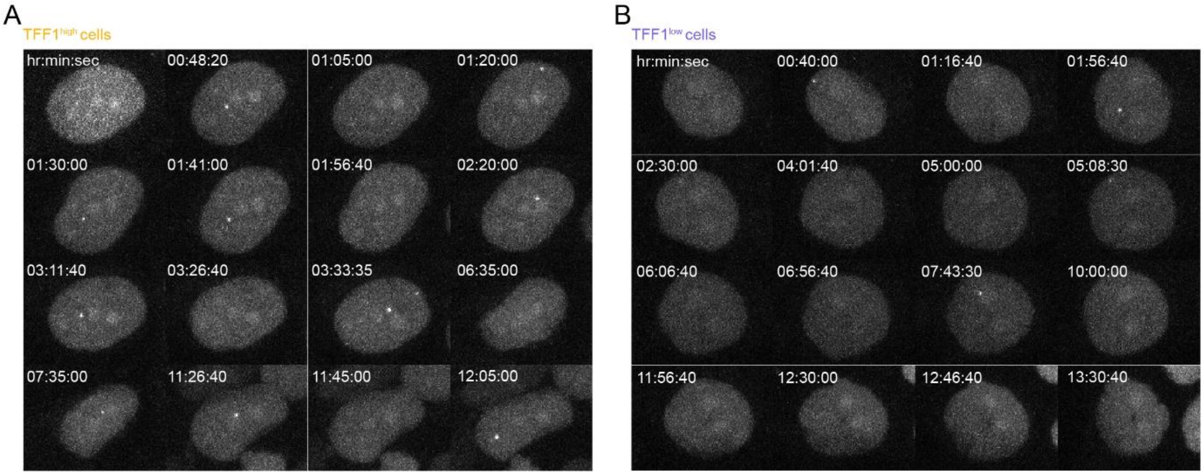

bioRxiv preprint doi: https://doi.org/10.1101/2025.09.18.677184; this version posted September 20, 2025. The copyright holder for this preprint (which was not certified by peer review) is the author/funder. This article is a US Government work. It is not subject to copyright under 17 USC 105 and is also made available for use under a CC0 license.

Supplementary Figure 4

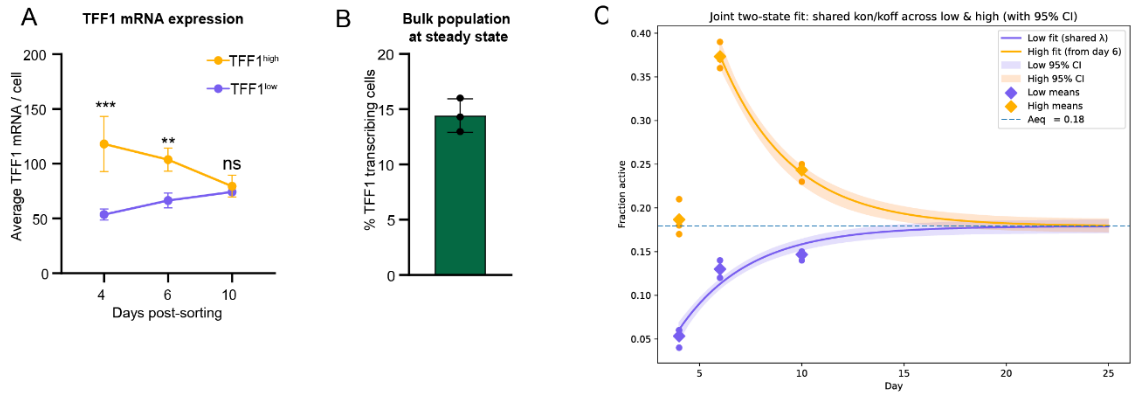

D

Šidák's multiple comparisons test	Mean Diff,	95,00% CI of diff,	Summary	Adjusted P Value
TFF1high vs. TFF1low				
Day 4	13,26	10,40 to 16,11	****	<0,0001
Day 6	24,3	21,45 to 27,15	****	<0,0001
Day 10	9,627	6,772 to 12,48	****	<0,0001

	Tukey's multiple comparisons test	Mean Diff,	95,00% CI of diff,	Summary	Adjusted P Value
TFF1high	Day 4 vs. Day 6	-18,65	-22,57 to -14,73	****	<0,0001
	Day 4 vs. Day 10	-5,613	-9,529 to -1,698	*	0,0109
	Day 6 vs. Day 10	13,04	9,121 to 16,95	***	0,0001

	Tukey's multiple comparisons test	Mean Diff,	95,00% CI of diff,	Summary	Adjusted P Value
TFF1low	Day 4 vs. Day 6	-7,607	-9,766 to -5,447	****	<0,0001
	Day 4 vs. Day 10	-9,243	-11,40 to -7,084	****	<0,0001
	Day 6 vs. Day 10	-1,637	-3,796 to 0,5229	ns	0,1276

bioRxiv preprint doi: https://doi.org/10.1101/2025.09.18.677184; this version posted September 20, 2025. The copyright holder for this preprint (which was not certified by peer review) is the author/funder. This article is a US Government work. It is not subject to copyright under 17 USC 105 and is also made available for use under a CC0 license.

Supplementary Figure 5

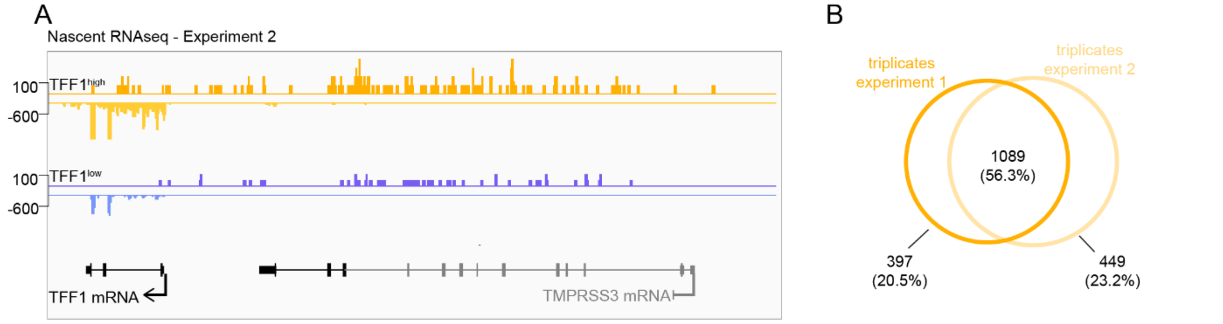

C

Estrogen Responsive genes upregulated in TFF1high cells

RET	INHBB	RASGRP1
SLC26A2	RHOD	CYP26B1
HSPB8	IL17RB	STC2
SEMA3B	AR	P2RY2
RHOBTB3	OLFM1	XBP1
ABAT	SYT12	EGR3
AREG	CHPT1	SIAH2
PRSS23	SLC27A2	ABCA3
TSKU	MSMB	ITPK1
RAPGEFL1	MYBL1	NPY1R
THSD4	MEST	PDZK1
CALB2	CAV1	CBFA2T3
JAK2	CPE	BCL2
IGFBP4	SLC29A1	TFF1
GREB1	BAG1	PTGES
KCNK15	KCNK5	SYNGR1

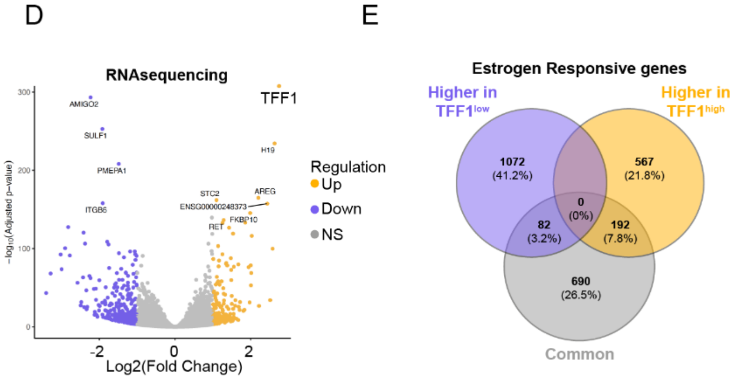

bioRxiv preprint doi: https://doi.org/10.1101/2025.09.18.677184; this version posted September 20, 2025. The copyright holder for this preprint (which was not certified by peer review) is the author/funder. This article is a US Government work. It is not subject to copyright under 17 USC 105 and is also made available for use under a CC0 license.

Supplementary Figure 6

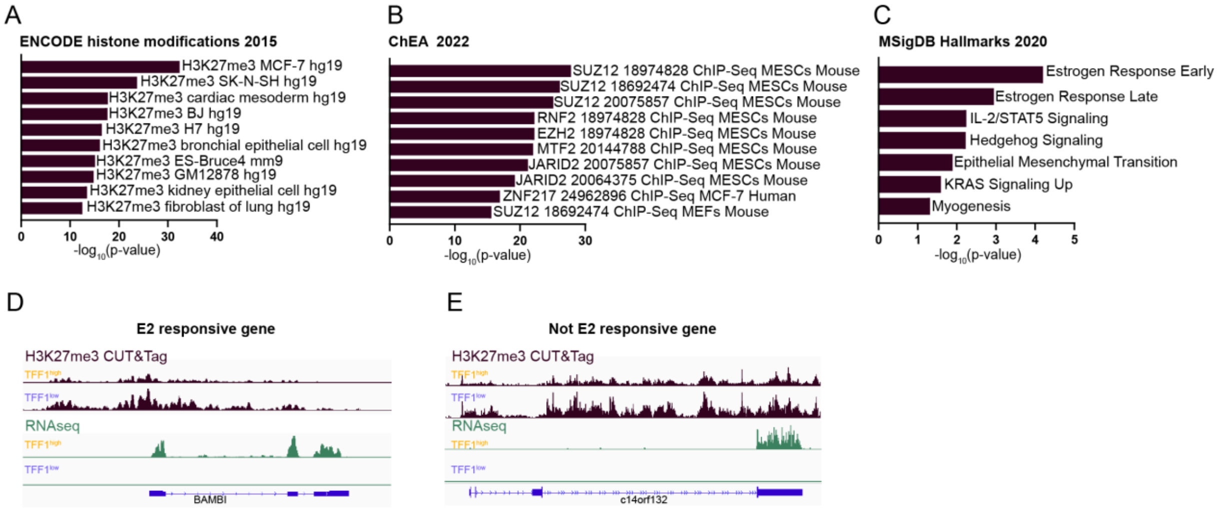

bioRxiv preprint doi: https://doi.org/10.1101/2025.09.18.677184; this version posted September 20, 2025. The copyright holder for this preprint (which was not certified by peer review) is the author/funder. This article is a US Government work. It is not subject to copyright under 17 USC 105 and is also made available for use under a CC0 license.

Table S2. Antibodies and smFISH probes

Antibody	Reference	Host	Application	Concentration
TFF1	#MAS-32781, Invitrogen	Rabbit	ImmunoFISH	1:200
Secondary anti-Rabbit AF50	#A-21039, CiteAb	Goat	ImmunoFISH	1:1000

Target	Probe Name	Sequence	Technology	Fluorescence
TFF1 exon	TFF1_exon_570_1	GAGGGACGTCGATGGTATTA	Stellaris	Cy3; 570nm
TFF1 exon	TFF1_exon_570_2	GTCAATCTGTGTTGTGAGCC	Stellaris	Cy3; 570nm
TFF1 exon	TFF1_exon_570_3	AGGAAAACCACAATTCTGTC	Stellaris	Cy3; 570nm
TFF1 exon	TFF1_exon_570_4	TTATTTGCACACTGGGAGGG	Stellaris	Cy3; 570nm
TFF1 exon	TFF1_exon_570_5	TTAGGCCAATTTTGAGTAGT	Stellaris	Cy3; 570nm
TFF1 exon	TFF1_exon_570_6	CGCAGATCACCTTGTTCTCC	Stellaris	Cy3; 570nm
TFF1 exon	TFF1_exon_570_7	CTAAAATTCACACTCCTCTT	Stellaris	Cy3; 570nm
TFF1 exon	TFF1_exon_570_8	TGGGACTAATCACCGTGCTG	Stellaris	Cy3; 570nm
TFF1 exon	TFF1_exon_570_9	TGTCGTCGAAACAGCAGC	Stellaris	Cy3; 570nm
TFF1 exon	TFF1_exon_570_10	GCCACTGTACACGTCTCT	Stellaris	Cy3; 570nm
TFF1 exon	TFF1_exon_570_11	GTCAGGATGCAGGCAGAT	Stellaris	Cy3; 570nm
TFF1 exon	TFF1_exon_570_12	TCTGAGGTGTCCGGTGGAG	Stellaris	Cy3; 570nm
TFF1 exon	TFF1_exon_570_13	TCTCTGCTCCAAAGGCGAC	Stellaris	Cy3; 570nm
TFF1 exon	TFF1_exon_570_14	GATAGAAGCACCAGGGGAC	Stellaris	Cy3; 570nm
TFF1 exon	TFF1_exon_570_15	CATGGACACCAGGACCAG	Stellaris	Cy3; 570nm
TFF1 exon	TFF1_exon_570_16	CACAGCTGCAGAAGCGT	Stellaris	Cy3; 570nm
TFF1 intron	TFF1_INTA_670_1	AGAGCAGGAAGAAGCACGCC	Stellaris	Cy5; 670nm
TFF1 intron	TFF1_INTA_670_2	GTAAAACAGTGGCTCCTGGCG	Stellaris	Cy5; 670nm
TFF1 intron	TFF1_INTA_670_3	ACAGCACCTGGCACAAAACA	Stellaris	Cy5; 670nm
TFF1 intron	TFF1_INTA_670_4	CCACCTGCTTTTGCCGATCC	Stellaris	Cy5; 670nm
TFF1 intron	TFF1_INTA_670_5	CTGCCACCCTGAGTTACTCT	Stellaris	Cy5; 670nm
TFF1 intron	TFF1_INTA_670_6	CGTCGCACTTCTCGAAGGTC	Stellaris	Cy5; 670nm
TFF1 intron	TFF1_INTA_670_7	AACAGCCCCGACTGAAGGCA	Stellaris	Cy5; 670nm
TFF1 intron	TFF1_INTA_670_8	ACATGACACTTGGGAGGATTG	Stellaris	Cy5; 670nm
TFF1 intron	TFF1_INTA_670_9	AGACCCCACGCCAACACTTC	Stellaris	Cy5; 670nm
TFF1 intron	TFF1_INTA_670_10	GGGAGATGTTGGCATGAACAG	Stellaris	Cy5; 670nm
TFF1 intron	TFF1_INTA_670_11	ATCTGGATGGGCCTTGGGA	Stellaris	Cy5; 670nm
TFF1 intron	TFF1_INTA_670_12	GCATTCGGGCCTGTGGGAGA	Stellaris	Cy5; 670nm
TFF1 intron	TFF1_INTA_670_13	AGGTGGCTTTGACTCCCAGA	Stellaris	Cy5; 670nm
TFF1 intron	TFF1_INTA_670_14	GTTGCCAGCCACGCACTCAT	Stellaris	Cy5; 670nm
TFF1 intron	TFF1_INTA_670_15	GGGCAACCGATCCACTTGAC	Stellaris	Cy5; 670nm
TFF1 intron	TFF1_INTA_670_16	CCACTCCCTAGAAGGACCCA	Stellaris	Cy5; 670nm
TFF1 intron	TFF1_INTA_670_17	AAGCAAGAATGGCCTCCTC	Stellaris	Cy5; 670nm
TFF1 intron	TFF1_INTA_670_18	AGTTGAGATGCAAACACTTCCC	Stellaris	Cy5; 670nm
TFF1 intron	TFF1_INTA_670_19	CTAGGGCAGACCGTTGATCC	Stellaris	Cy5; 670nm
TFF1 intron	TFF1_INTA_670_20	GCCACACAGCATTTCCTGAC	Stellaris	Cy5; 670nm
TFF1 intron	TFF1_INTA_670_21	AAGTGCAAGTCGCAGATGCT	Stellaris	Cy5; 670nm
TFF1 intron	TFF1_INTA_670_22	ATAAGTTATTCAGCTCCCCACAG	Stellaris	Cy5; 670nm
TFF1 intron	TFF1_INTA_670_23	CCCCACCTTGAAACTGTACCT	Stellaris	Cy5; 670nm
TFF1 intron	TFF1_INTA_670_24	ACCCTTGCTTTGGAAACGTAG	Stellaris	Cy5; 670nm
TFF1 intron	TFF1_INTA_670_25	GCGCTCCCTCTTCAGGCCTC	Stellaris	Cy5; 670nm
TFF1 intron	TFF1_INTA_670_26	GGGCACGGACAGCACCACT	Stellaris	Cy5; 670nm
TFF1 intron	TFF1_INTA_670_27	AGGTGGCTGGAGGGCAGTGG	Stellaris	Cy5; 670nm
TFF1 intron	TFF1_INTA_670_28	CCGGTACCCCACAGCAGAGA	Stellaris	Cy5; 670nm
TFF1 intron	TFF1_INTA_670_29	CTGGCTGCTCTTCTACGCTG	Stellaris	Cy5; 670nm
TFF1 intron	TFF1_INTA_670_30	GGTCTTCCCACTGCAGCCC	Stellaris	Cy5; 670nm
TFF1 intron	TFF1_INTA_670_31	CTCTCGGGTTCCAAAGCTCC	Stellaris	Cy5; 670nm
TFF1 intron	TFF1_INTA_670_32	CGTTCTGTACACCGAGGCCA	Stellaris	Cy5; 670nm
TFF1 intron	TFF1_INTA_670_33	CCAGCACGGGGACCCACCCA	Stellaris	Cy5; 670nm
TFF1 intron	TFF1_INTA_670_34	CAAGGGCGCAGGCAGATGGG	Stellaris	Cy5; 670nm
TFF1 intron	TFF1_INTA_670_35	CCACGGCCTCTGTGATCCCA	Stellaris	Cy5; 670nm
TFF1 intron	TFF1_INTA_670_36	GCCTTGAGCCAGAGGGTGTG	Stellaris	Cy5; 670nm
TFF1 intron	TFF1_INTA_670_37	AGTGACCCGCAAAGACGCTC	Stellaris	Cy5; 670nm
TFF1 intron	TFF1_INTA_670_38	ATGGTTTCCACACACGAATGC	Stellaris	Cy5; 670nm
TFF1 intron	TFF1_INTA_670_39	TCTTGCCCACCCGCTTTGTC	Stellaris	Cy5; 670nm
TFF1 intron	TFF1_INTA_670_40	GAAGATTTCCAGCCCCGGTG	Stellaris	Cy5; 670nm
TFF1 intron	TFF1_INTA_670_41	GCCCCGTGGTGAGGGAGGAT	Stellaris	Cy5; 670nm
TFF1 intron	TFF1_INTA_670_42	CGACGTGAAGGTGATCATCGC	Stellaris	Cy5; 670nm
TFF1 intron	TFF1_INTA_670_43	CATGGGAACCTTGCCCTCCT	Stellaris	Cy5; 670nm
TFF1 intron	TFF1_INTA_670_44	GGATCCGTGTTCAGCTCCCG	Stellaris	Cy5; 670nm
TFF1 intron	TFF1_INTA_670_45	GGAGAAAGTGTTCCTGGCTTG	Stellaris	Cy5; 670nm
TFF1 intron	TFF1_INTA_670_46	ATCAGGGACTGCCTCGAGAT	Stellaris	Cy5; 670nm
TFF1 intron	TFF1_INTA_670_47	GGGCTGTTCTCTTGTCTGCT	Stellaris	Cy5; 670nm
TFF1 intron	TFF1_INTA_670_48	CCTTGGGGCATAGGAGGGGA	Stellaris	Cy5; 670nm
TFF1 intron	TFF1_INTB_670_1	GCCCAGAGCACCCTCCGGTC	Stellaris	Cy5; 670nm
TFF1 intron	TFF1_INTB_670_2	AGGATCTGGTGGTTGACAGC	Stellaris	Cy5; 670nm
TFF1 intron	TFF1_INTB_670_3	CGCGGAATCAAAGGTCTCAGA	Stellaris	Cy5; 670nm
TFF1 intron	TFF1_INTB_670_4	AGGTTCTAACACCAGAGCTTCA	Stellaris	Cy5; 670nm
TFF1 intron	TFF1_INTB_670_5	GGGCTGCCGCAGGATCTTAT	Stellaris	Cy5; 670nm
TFF1 intron	TFF1_INTB_670_6	ACGTGGACAGATGGGGCTGG	Stellaris	Cy5; 670nm
TFF1 intron	TFF1_INTB_670_7	TGAAGCAGGGCAGTGAGGAA	Stellaris	Cy5; 670nm
TFF1 intron	TFF1_INTB_670_8	AGGAGCCGACAGAGGGTACA	Stellaris	Cy5; 670nm
TFF1 intron	TFF1_INTB_670_9	TGAGAGGACCGGGTGGGGTG	Stellaris	Cy5; 670nm
TFF1 intron	TFF1_INTB_670_10	AGGAGGTGGAAACCCATTGC	Stellaris	Cy5; 670nm
TFF1 intron	TFF1_INTB_670_11	CATTCCAGAGACCCAGGTGT	Stellaris	Cy5; 670nm
TFF1 intron	TFF1_INTB_670_12	CCAGGTGAAGATGGAGCCGC	Stellaris	Cy5; 670nm
TFF1 intron	TFF1_INTB_670_13	AGGCCAATGACAAGCAGGTT	Stellaris	Cy5; 670nm
TFF1 intron	TFF1_INTB_670_14	GACAACGCAAGGCCATCGTC	Stellaris	Cy5; 670nm
TFF1 intron	TFF1_INTB_670_15	AGAAAGGCAAGAACATGTGCG	Stellaris	Cy5; 670nm
TFF1 intron	TFF1_INTB_670_16	AGAGTCTAAGGATCTGATGGAGG	Stellaris	Cy5; 670nm
TFF1 intron	TFF1_INTB_670_17	CCAGCCTGAGTGACAGAGCA	Stellaris	Cy5; 670nm
TFF1 intron	TFF1_INTB_670_18	ATCACTTGAACCCAGGAGGCA	Stellaris	Cy5; 670nm
TFF1 intron	TFF1_INTB_670_19	TACTTGGGAGGCTGAGGCAGGAG	Stellaris	Cy5; 670nm
TFF1 intron	TFF1_INTB_670_20	AAATTAGGCGGGCATTGTGG	Stellaris	Cy5; 670nm
TFF1 intron	TFF1_INTB_670_21	AGACCAGCCTGACCAAAAT	Stellaris	Cy5; 670nm
TFF1 intron	TFF1_INTB_670_22	ACTTTGGGAGGCTGAAGGAGGT	Stellaris	Cy5; 670nm
TFF1 intron	TFF1_INTB_670_23	ACTGTGCCAGGTGAGAGAG	Stellaris	Cy5; 670nm
TFF1 intron	TFF1_INTB_670_24	TGTGCATGAGAAGTGGGAGC	Stellaris	Cy5; 670nm
TFF1 intron	TFF1_INTB_670_25	TGCCCCAGGTTTGTGGACTT	Stellaris	Cy5; 670nm
TFF1 intron	TFF1_INTB_670_26	GTGCAAACATTACGCTCAGGG	Stellaris	Cy5; 670nm
TFF1 intron	TFF1_INTB_670_27	GGGAGACGTGGTCCTCACAT	Stellaris	Cy5; 670nm
TFF1 intron	TFF1_INTB_670_28	CCAGTGAGGCGGATATAAAACC	Stellaris	Cy5; 670nm
TFF1 intron	TFF1_INTB_670_29	CACACCCATCCAGCTTCCTT	Stellaris	Cy5; 670nm
TFF1 intron	TFF1_INTB_670_30	AGTTGTGGGCTGAATTCCTT	Stellaris	Cy5; 670nm
TFF1 intron	TFF1_INTB_670_31	AAAGTGCACAGGTAAGAAGCAA	Stellaris	Cy5; 670nm

bioRxiv preprint doi: https://doi.org/10.1101/2025.09.18.677184; this version posted September 20, 2025. The copyright holder for this preprint (which was not certified by peer review) is the author/funder. This article is a US Government work. It is not subject to copyright under 17 USC 105 and is also made available for use under a CC0 license.
